# Naked mole rat cells display more efficient excision repair than mouse cells

**DOI:** 10.18632/aging.101482

**Published:** 2018-06-20

**Authors:** Alexei Evdokimov, Mikhail Kutuzov, Irina Petruseva, Natalia Lukjanchikova, Elena Kashina, Ekaterina Kolova, Tatyana Zemerova, Svetlana Romanenko, Polina Perelman, Dmitry Prokopov, Andrei Seluanov, Vera Gorbunova, Alexander Graphodatsky, Vladimir Trifonov, Svetlana Khodyreva, Olga Lavrik

**Affiliations:** 1 Institute of Chemical Biology and Fundamental Medicine, Novosibirsk 630090, Russia; 2 Institute of Molecular and Cellular Biology, Novosibirsk 630090, Russia; 3 Institute of Cytology and Genetics, Novosibirsk 630090, Russia; 4 Department of Biology, University of Rochester, Rochester, NY 14627, USA; * Equal contribution

**Keywords:** base excision repair, *Heterochephalus glaber*, Mus musculus, nucleotide excision repair, poly(ADP-ribose) polymerase 1

## Abstract

Naked mole rat (NMR) is the long-lived and tumor-resistant rodent. NMRs possess multiple adaptations that may contribute to longevity and cancer-resistance. However, whether NMRs have more efficient DNA repair have not been directly tested. Here we compared base excision repair (BER) and nucleotide excision repair (NER) systems in extracts from NMR and mouse fibroblasts after UVC irradiation. Transcript levels of the key repair enzymes demonstrated in most cases higher inducibility in the mouse vs the NMR cells. Ratios of repair enzymes activities in the extracts somewhat varied depending on post-irradiation time. NMR cell extracts were 2–3-fold more efficient at removing the bulky lesions, 1.5–3-fold more efficient at removing uracil, and about 1.4-fold more efficient at cleaving the AP-site than the mouse cells, while DNA polymerase activities being as a whole higher in the mouse demonstrate different patterns of product distribution. The level of poly(ADP-ribose) synthesis was 1.4–1.8-fold higher in the NMR cells. Furthermore, NMR cell extracts displayed higher binding of PARP1 to DNA probes containing apurinic/apyrimidinic site or photo-reactive DNA lesions. Cumulatively, our results suggest that the NMR has more efficient excision repair systems than the mouse, which may contribute to longevity and cancer resistance of this species.

## Introduction

Aging and cancer are accompanied by the accumulation of mutations in the genome, genomic instability and dysregulation of transcription patterns [1]. DNA repair systems have evolved to counteract genomic instability. However, whether long-lived and cancer-resistant animal species have more efficient DNA repair is unclear. Comparative studies of nucleotide excision repair (NER) provided contradicting results [2], while one study that measured base excision repair in several species did not find correlation with lifespan [3].

Naked mole rat (NMR), *Heterocephalus glaber*, is the longest-lived rodent with the maximum lifespan of 32 years [4], which is almost ten times longer than a house mouse. Furthermore, NMRs are resistant to cancer [5,6] with spontaneous tumors being extremely rare [7]. NMRs evolved a variety of adaptations that may contribute to longevity and cancer resistance [8–12]. Some of these adaptations may promote genome and proteome stability and increase resistance to stress. NMR proteins involved in redox processes are more resistant to denaturing agents and are able to maintain function under oxidative stress [13,14]. High accuracy of translation process [15], increased level of expression of key chaperones and more active proteasomes [16] help to maintain a pool of functional proteins. Transcriptome analyses by RNA sequencing showed that several genes involved in DNA repair are up-regulated in *H. glaber* cells [17,18]. However, transcript levels do not always unambiguously reflect the level of protein expression and activity [19]. NMR cells were found to be more resistant than mouse cells to a variety of stressors such as cadmium ions, MMS, paraquat, heat, and low glucose media [20]. Intriguingly, despite the up-regulated expression of some BER and NER related genes [17,18], NMR fibroblasts were more sensitive to H_2_O_2_ and UV light [20]. Cell survival under stress is a function of the repair capacity, cell cycle checkpoints, and apoptotic responses. Therefore, NMRs may have more efficient BER and NER systems that protect the cells from mutations coupled with heightened stress responses. Here we performed the analysis of BER and NER systems in NMR and mouse (*Mus musculus*) fibroblasts in response to UVC-light exposure. We evaluated post-irradiation changes in mRNA transcription of several key reparative proteins and measured the activities of the key BER and NER enzymes. One of the immediate cellular responses to DNA damage is poly(ADP-ribosyl)ation, i.e., the transient covalent modification of proteins by a homopolymer consisting of ADP ribose units. This reaction is catalyzed by poly(ADP-ribose)polymerases (PARPs), which are activated by DNA damage. PARPs and their activity are thought to act as an essential system for regulation of several DNA repair systems including BER and NER [21–24]. In addition, PARPs activity correlates with mammalian life span [24]. Analysis of poly(ADP-ribosyl)ation synthesis catalyzed by endogenous poly(ADP-ribose) polymerases of NMR and mouse cell extracts revealed more active PARylation in NMR cells. Our results suggest that NMR has more efficient BER and NER systems than the short-lived and tumor-prone mouse.

## RESULTS

**Figure 1:**
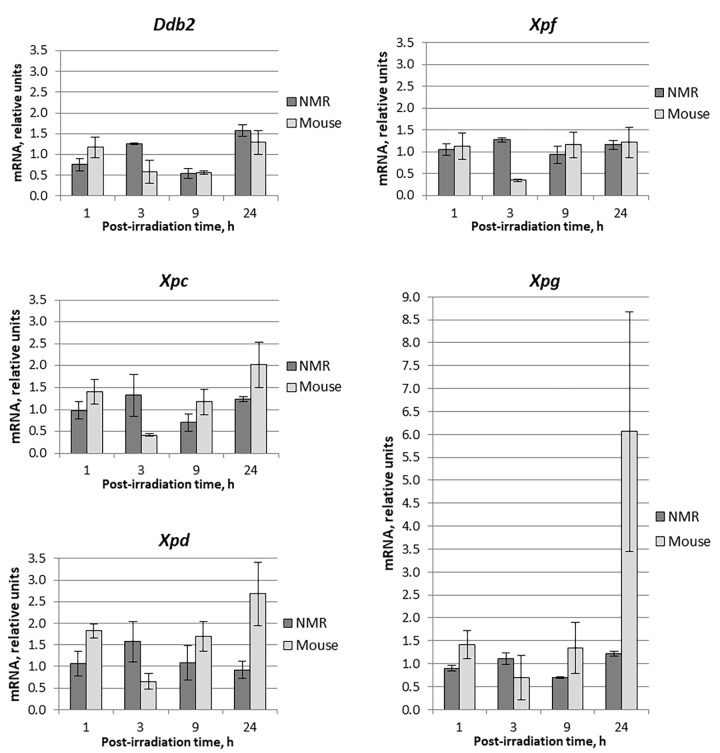
**Time dependent levels of mRNA encoding NER proteins in NMR and mouse cells after UVC-light irradiation.** Time of cells incubation after UVC-light irradiation is presented on the X-axis. For each gene, the level of its expression in UVC-light irradiated cells was normalized to that in untreated cells with *Tubb2a, Tubb1, Polr1b, and Actb* genes used for reference. Experiments were repeated at least three times; mean values ± SD are shown.

To characterize the response of DNA repair systems to exposure to UVC irradiation, we analyzed the functionality of the BER and NER systems in NMR and mouse fibroblasts using several approaches and tested cell proliferation after UVC irradiation. UVC-light is known to generate predominantly DNA lesions, which are specifically recognized and removed by the NER proteins [25]. However, it has been found that UVC-light exposure induces also oxidation of nucleobases via a direct process (one-electron oxidation) ([26] and references therein). The activity of the BER system is indispensable to remove these lesions [25]. Analysis of metabolic activity/viability of cells at the time points after irradiation that then were used for the tests of enzymatic activities and evaluation of mRNA levels (Fig. 1S) revealed neither significant variations of the metabolic activity nor the difference between NMR and mouse cells; this indirectly testifies to comparable sensitivity of the cells to UVC-irradiation.

### mRNA expression

First, using RT-qPCR method we compared the relative content of mRNAs encoding NER and BER proteins in mouse and NMR cells at different time intervals after UVC-light exposure. Data for the NER-related proteins – DDB2, XPC, XPD, XPF, and XPG – are shown in Fig. 1.

The analysis revealed that mRNA content fluctuations (decrease or increase) did not exceed twofold, especially for NMR. The exceptions were XPC-, XPD- and XPG-coding mRNAs, the relative content of which in mouse cells at 24 hours of post-UVC irradiation the content increased 2, 2.7 and 6 times, respectively.

In NMR cells, mRNA levels of the NER genes showed little change after UVC irradiation (Fig. 1). A mild increase in mRNA levels occurred after 3 hours (1.1–1.6 fold), followed by a decrease to the basal levels and even lower after 9 hours (0.5-1.1 fold). In mouse cells, mRNA levels showed more profound change, where an increase at 1 hour after UV- irradiation (for Xpd up to 1.7 fold) followed by a decrease at 3 hours (to about half of the control level). Then the mRNA content for XPC, XPD and XPG began to increase and at 24 hours exceeded several times the control level (2.5, 3, and 7-fold, respectively) (Fig. 1). This is consistent with earlier reports that *Xpg* expression increased following UV-exposure in mouse fibroblasts [27].

**Figure 2:**
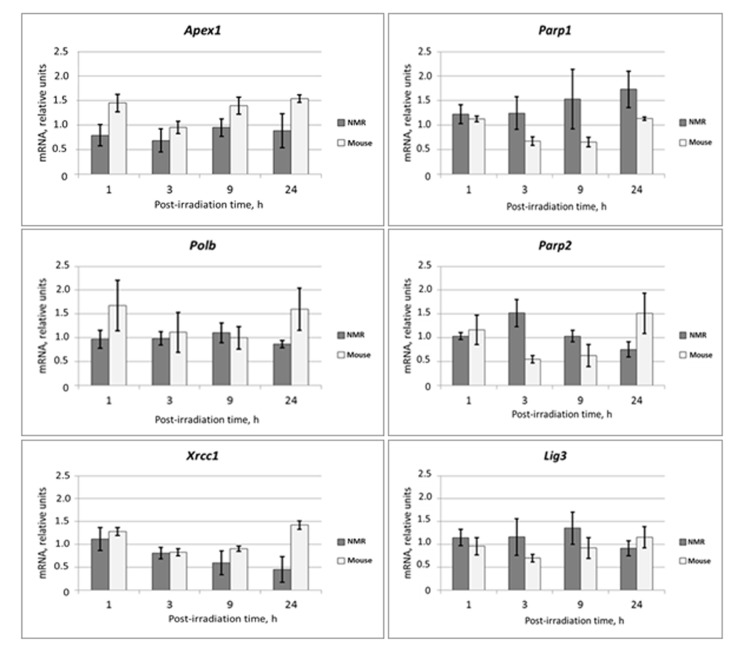
**Time dependent levels of mRNA encoding BER proteins in NMR and mouse cells after UVC-light irradiation.** The data are the mean of three independent experiments made in triplicates ±SD. For each gene the level of its expression in UVC-irradiated cells was normalized to that of non-irradiated cells. Three housekeeping genes: *Tubb, Actb* and *Gapdh* were used as a reference.

The mRNA levels of theBER genes *Apex1, Parp1, Parp2, Xrcc1, pol b*, and *Lig 3* were analyzed in the same mRNA samples described above (Fig. 2).

mRNA levels did not change more than two-fold for neither of the mouse BER genes. For most mouse genes, mRNA levels dropped at the 3 and 9 h points followed by an increase at the 24 h point. For NMR cells, the temporal decrease in mRNA levels at the 3 and 9 h points was not characteristic; the levels of mRNAs at 24 h point in most cases except *Parp1* were comparable or even lower than that in non-irradiated cells. Interestingly, mRNA levels of the two regulatory BER proteins, XRCC1 and PARP1, changed in opposite directions. The level of the XRCC1 mRNA smoothly decreased from the 1 h to 24 h time point while that for PARP1 steadily increased. These results indicate that mRNA levels for NER and BER proteins show a tendency to increase following UVC-irradiation in mouse cells, but not in the NMR cells. In summary, the changes in mRNA levels following UVC-irradiation were considerably lower in NMR than in the mouse cells.

### Excision activity of the NER system

NER process involves several steps: primary recognition, verification, and excision of DNA fragments containing bulky lesions. To characterize the efficiency of the different stages of the NER process in NMR and mouse cells we employed an assay based on post-excision labelling of the removed DNA fragment. The flowchart of the assay is shown in Fig. S6. The 137-mer DNA duplex containing non-nucleotide insertion – N-[6-(5(6)-fluoresceinylcarbamoyl)hexanoyl]-3-amino-1,2-propandiol (nFlu-DNA) – has been previously shown to be an efficient NER-substrate [28]. Pilot experiments revealed that specific excision of nFlu-containing DNA fragment was significantly more efficient in the extracts from NMR fibroblasts than in the extracts of mouse fibroblasts derived from either untreated cells or cells incubated for 24 h after UVC-irradiation (Fig. S2). We next set out to investigate the excision repair process in more detail.

**Figure 3:**
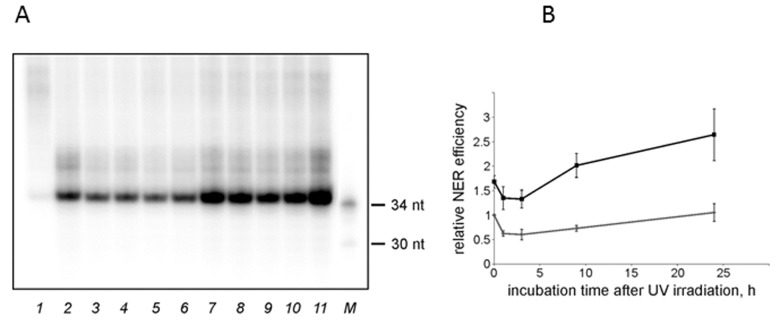
**The NER excision activity of NMR and mouse cell extracts.** (**A**) Representative phospho-image of product analysis for mouse (lanes 2-6) and NMR (lanes 7-11) cell extracts. The substrate DNA was incubated for 45 min at 30°C with cell extracts (15 nM substrate DNA, 0.3 mg/ml extract proteins). The excision products were detected by annealing to a specific template containing 5ʹ-GpGpGpGpG overhang, which was then end-labeled using α-[^32^P]-dCTP and Taq DNA polymerase. The reaction products were resolved on a 10% denaturing polyacrylamide gel. [^32^P]-5ʹ end-labelled oligonucleotides were used as length markers (lane M). nFlu-DNA without cell extract was used as a negative control (lane 1). (**B**) Quantification of the levels of excision products based on three independent experiments. The activity in the extract of mouse non-irradiated cells was taken as 1. The data are the mean ±SD, n=3. Grey and black lines correspond to mouse and NMR cell extracts, respectively.

Based on the time course for the NER excision activity (Fig. S4), we chose 45 min time point for further analysis. The kinetics of the excision reaction was similar in the NMR and the mouse cell extracts. The incision activity dropped at 1-3 h post-irradiation, followed by an increase in specific excision until complete recovery. In the NMR cell extract the activity recovered faster and reached basal levels at 9 h post irradiation, while in the mouse extract it completely recovered only after 24 h (Fig. 3). Moreover, in the NMR cell extract, the activity at 24 h post-irradiation point was higher than in the extract of non-irradiated cells.

These experiments suggest that both basal and UVC-induced NER excision activity is higher in the NMR than in the mouse cells. Interestingly, in both species post-irradiation changes in the NER excision activity did not correlate with the levels of mRNAs encoding the NER proteins. Moreover, at 24 h post-irradiation mouse cells demonstrated higher amplitude of response at the level of mRNA than NMR cells.

### Activity of the BER system

**Figure 4:**
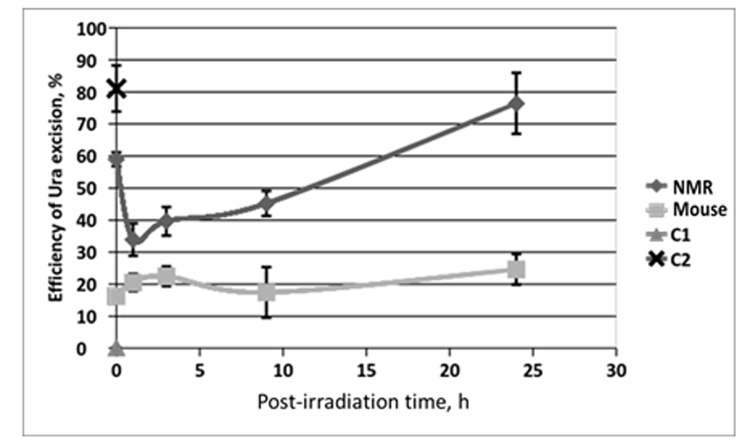
**The efficiency of uracil excision from DNA by extracts from NMR and mouse cells with different post-irradiation time.** Reaction mixtures contained 100 nM 5'-[^32^P] 32-mer U-DNA, 50 mM Tris-HCl (pH 8.0), 40 mM NaCl, 10 mM EDTA, 1 mM DTT, 0.1 mg/ml BSA and 0.5 mg/ml of cell extract proteins. The reaction mixtures were incubated at 37^0^C for 10 min. C1 – control reaction mixture contained only U-DNA. C2 – control reaction mixture corresponds to U-DNA incubated with excess of *E. coli* UDG. Then reaction mixtures were treated and analyzed as described in the section “Uracil excision activity of cell extracts”. Efficiency of Ura removal is determined as percent of cleaved Ura containing DNA chain.

To compare the efficiency of the BER pathway in NMR and mouse, we examined the activity of several key BER proteins in the extracts of NMR and mouse cells. First, we determined the efficiency of uracil (Ura) excision from dsDNA. Uracil residues appear in genomic DNA via two ways: cytosine deamination or misincorporation of dUMP residues. This DNA lesion is removed via short-patch BER, initiated by monofunctional uracil DNA glycosylase (UDG) or other DNA glycosylases – TDG and MBD4 – which are also able to remove Ura residues, although less efficiently [29,30]. UDG excises uracil in both A:U and G:U pairs. To estimate the efficiency of Ura removal by glycosylases, Ura containing DNA duplex (U-DNA) was incubated with extracts of NMR and mouse cells. In control reaction mixture U-DNA was incubated with excess of *E. coli* UDG. The resulting AP DNA was then cleaved at AP site by heating under alkaline conditions and analyzed by PAG-7 M urea electrophoresis. The efficiency of Ura excision is presented in Fig. 4.

In all cases, a more efficient excision of Ura was observed in the NMR cell extracts as compared to the mouse ones. For the NMR cell extracts, a considerable drop of Ura removal efficiency occurred at 1 h after irradiation followed by gradual recovery to the initial level characteristic for non-irradiated cells (about 16 h post-irradiation time) and further increased exceeding basal level at 24 h after irradiation. On the contrary, no drop below basic level was observed in mouse cells. For both type of cells, Ura removal activity at 24 h after irradiation was higher in comparison with basic level in untreated cells, with relative increment being comparable. The ratios of activity at 24 h point versus that in untreated cells were 1.3 and 1.5 for NMR and mouse cell extracts, respectively. It should be noted, that the higher basic level of Ura removal activity in NMR versus mouse cells may be related with the increased level of expression of TDG and MBD4 glycosylases, which was observed in NMR liver cells [17]. These DNA glycosylases are also capable of Ura removal, particularly in U:G pairs (as in the substrate used here) [29–31].

**Figure 5:**
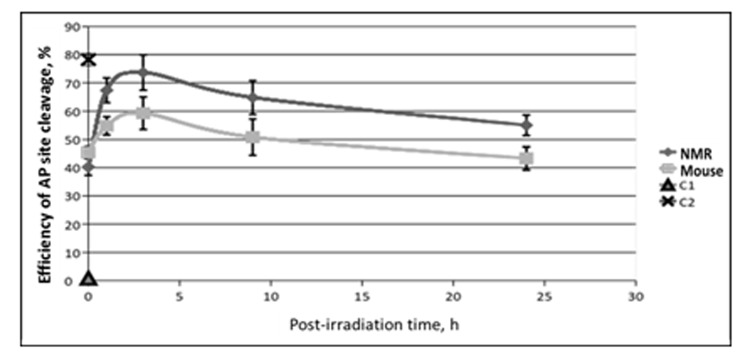
**The efficiency of AP site cleavage by extracts from NMR and mouse cells with different post-irradiation time.** Reaction mixtures contained 100 nM 5'-[^32^P] 32-mer AP DNA, 0.5 mg/ml of cell extract and buffer components. The reaction proceeded at 37ºC for 10 min and then was quenched by addition of EDTA (20 mM final) followed by stabilization of intact AP sites with NaBH_4_ treatment. DNA was then analyzed as described in the section ‘AP site cleavage activity of cell extracts’**.** C1 – incubation of AP DNA in the absence of cell extracts reflects the level of spontaneous AP site cleavage. C2 – incubation of AP DNA with 100 µM NaOH that brings about full AP site cleavage.

AP sites, a central intermediate in the BER process, are generated by various DNA glycosylases or occur spontaneously [32,33]. AP sites are most abundant among DNA lesions; their level is estimated as 50,000 lesions per cell per day [34]. In mammals the AP site repair is mainly initiated by AP endonuclease 1 (APE1), the second enzyme of the canonical BER pathway [34]. An additional pathway of AP sites repair is to cleave them by bifunctional DNA glycosylases and other enzymes with associated AP lyase activity [33,35]. In this case, the Schiff base intermediate between the C1′ atom of deoxyribose and a primary amine group in the protein is formed. Recently it has been found in *in vitro* system that tyrosyl-DNA-phosphodiesterase 1 is also able to cleave AP sites and initiate an alternative BER pathway [36]. The efficiency of AP site cleavage in NMR and mouse cell extracts was tested using the AP site containing DNA duplex (32 bp). AP sites in DNA were generated by treatment with *E. coli* UDG immediately before the experiment. The results are shown in Fig. 5.

The basic levels of AP site cleavage were equal in the extracts of untreated NMR and mouse cells. This result is in accordance with previous reports showing equal levels of APE1 mRNA in liver cells of NMR and mouse [17]. Interestingly, unlike activity of the NER system and Ura removal, the efficiency of AP site cleavage in the extracts of irradiated cells, never dropped below the basal levels characteristic of non-irradiated cells in both NMR and mouse. For both cell lines the highest increment of AP site cleavage activity above the basal level occurred at 3 h point followed by a slow decrease. For mouse cells at 24 h point the activity returned to the basal level, while for NMR cells it was still more than 30% higher comparatively the basal value. It should be noted that the test used here could not discriminate AP site cleavage by APE1 and bifunctional DNA glycosylases, but APE1 is known as the main AP site processing enzyme in mammalian cells [37], its activity is several orders of magnitude higher than that of bifunctional DNA glycosylases. In summary, NMR cells demonstrated a stronger response to treatment with UVC-light than mouse cells.

To compare the efficiency of reparative DNA synthesis in the NMR and mouse cell extracts we used a [^32^P]-labeled 32-mer DNA duplex with one nucleotide gap flanked at the 3' end by hydroxyl group and at the 5' end either by deoxyribose phosphate (dRP) or diethyleneglycol phosphate (pDEG) group.

DNA with 5'-dRP moiety is a natural DNA intermediate of short patch (SP) BER while DNA with pDEG can be considered an intermediate of long patch (LP) BER, since pDEG group is refractory to the deoxyribose phosphate lyase activity of Polβ. Polβ known as a main DNA polymerase of SP BER displays two activities: it incorporates dNMP by its deoxynucleotidyl transferase activity and removes the 5'-dRP moiety due to intrinsic deoxyribose phosphate lyase activity thereby generating the 5' end phosphate for the following ligation step [33].

**Figure 6:**
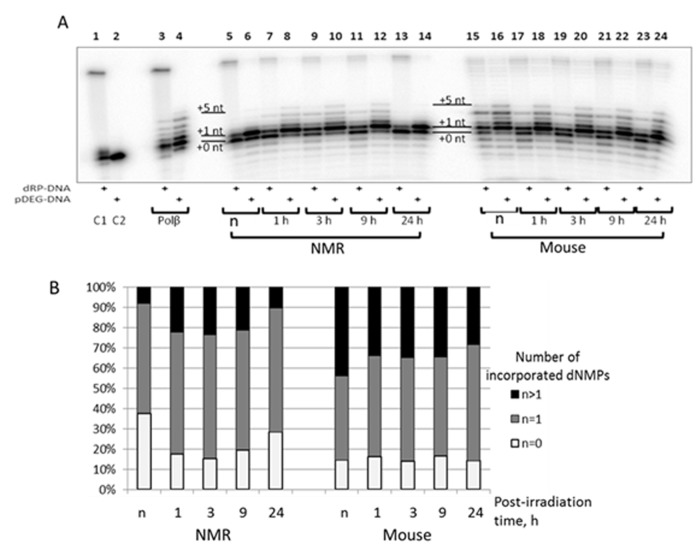
**The efficiency of DNA synthesis by extracts from NMR and mouse cells with different post-irradiation time.** (**A**) The 100 nM 5'-[^32^P] 32-mer DNA with 5'-dRP-nick (odd lanes) or with 5'-pDEG-nick (even lanes) were incubated with 0.1 µM Polβ (lanes 3 and 4), 0.5 mg/ml of cell extract protein of NMR (lanes 5 – 14) or mouse (lanes 15 – 24) fibroblasts and buffer components. The reaction mixtures were incubated at 37ºC for 10 min. The control probes C1 and C2 contained only substrate DNAs and buffer components. The mixtures were supplemented with loading buffer, heated at 97^0^C for 10 min, and the products were then analyzed as described in ‘DNA polymerase activity of cell extracts**’.** (**B**) Quantification of the DNA synthesis products for 5'-pDEG-nick DNA shown in A. White parts of bars correspond to non-elongated primer, grey parts reflect the amount of primer elongated by one dNMP and black parts correspond to the products of strand-displacement DNA synthesis. ‘n’ designates non-irradiated cells.

The SP BER substrate was obtained by treatment of AP-site containing DNA by APE1 immediately before experiment because of instability of 5'-dRP moiety. The products of DNA synthesis by Polβ on the substrates of SP and LP BER are represented in Fig. 6, lanes 3 and 4, respectively. DNA polymerase β efficiently inserts one nucleotide (gap-filling stage) followed by strand-displacement DNA synthesis (insertion of several dNMPs); and is more efficient on DNA substrate of LP pathway. Analogous patterns have been demonstrated earlier [38,39]. The 10 min point for the reaction of DNA synthesis was chosen as a compromise combining a robust detection of DNA synthesis products with an acceptable level of primer degradation by intrinsic nucleases of the extracts. Post-irradiation time dependence of DNA synthesis in the extracts demonstrates quite different patterns. For the NMR cell extract, a well-marked increase in the efficiency of primer elongation occurred at 1, 3, and 9 h points post-irradiation as compared with non-irradiated cells (lanes 7-12 versus lanes 5 and 6); at 24 h the efficiency dropped to the initial value (lanes 13 and 14 versus lanes 5 and 6), while for mouse cells no considerable post-irradiation-time-dependent change was observed (lanes 17-24 versus lanes 15 and 16). It should be stressed that the pattern of the products resembles those for purified Polβ. In addition, for the mouse cell extract, low levels of longer products (up to incorporated 15-16 dNMPs) were detected. Distribution of DNA synthesis products on substrate of LP pathway (panel B) indicates that the mouse cell extracts are more efficient at strand-displacement DNA synthesis. In summary, DNA synthesis is more efficient in mouse cell extract taking into account smaller portion of non-elongated primer (Fig. 6B, white bars) and the amounts of primer elongated for more than one nucleotide (Fig. 6B, black bars). The products of gap filling DNA synthesis (incorporation of one nucleotide) (Fig. 6B, grey bars) comprise about 60% for NMR and 50% for mouse cell extracts, respectively. The dynamics of DNA synthesis observed in NMR cell extracts does not correlate with the levels of mRNA encoding DNA polymerase β (Fig. 2). It should be noted that Polβ was not among the proteins with increased expression in NMR versus mouse liver cells [17].

### Activity of the PARylation system

Posttranslational modification of cellular proteins with poly(ADP-ribose) (PAR) is one of the immediate key reactions to DNA damage in cells of higher eukaryotes. Using NAD^+^ as substrate, poly(ADP-ribose) polymerases catalyze synthesis of (ADP-ribose) polymer covalently attached to target proteins including PARP themselves. Three of PARPs —PARP1, PARP2, and PARP3—play a role in the cell response to DNA damage being activated upon interaction with damaged DNA [21–23]. PARPs and their activity are thought to act as an essential system for regulation of several DNA repair systems including BER and NER [21–23].

**Figure 7:**
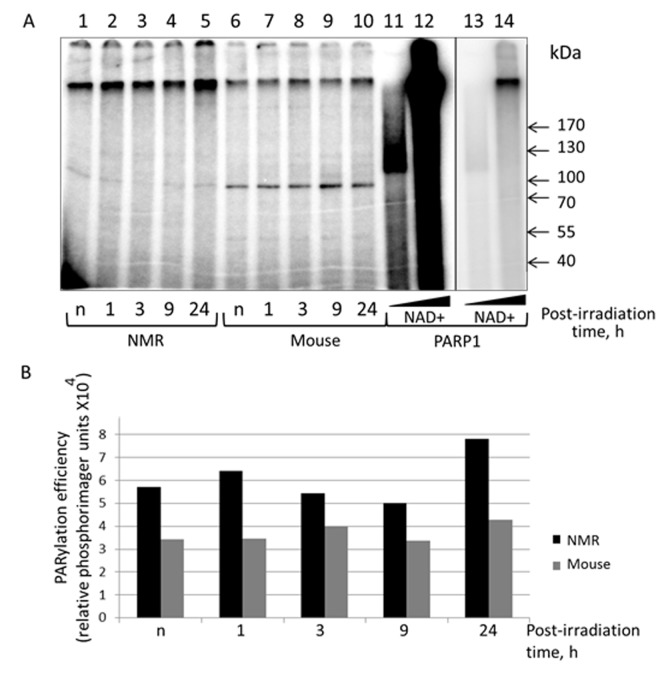
**PARylation of cell extract proteins of NMR and mouse cells in presence of [^32^P]NAD^+^.** (**A**) The activated DNA was incubated with 0.5 mg/ml of cell extracts of NMR cells (lanes 1 – 5) or mouse cell (lanes 6 – 10). The control probes (lanes 11–14) contained 70 nM PARP1 instead of cell extracts and 40 µM [^32^P]NAD^+^ (lanes 11 and 13) or 400 µM [^32^P]NAD^+^ (lanes 12 and 14). Lanes 13 and 14 correspond to lanes 11 and 12 but with low exposure. The reaction mixtures were incubated at 37^0^C for 4 min. The mixtures were supplemented with loading buffer, heated at 97^0^C for 10 min and then analyzed as described in the section ‘PARylation of cell extract proteins’. (**B**) Quantification showing the yield of PARylation products (**A**). Post-irradiation time is the time the cells were cultivated after irradiation before the cells were harvested for the following extract preparation. “n” designates non-irradiated cells.

To further characterize the response of NMR and mouse cells to UVC irradiation we tested the intensity of poly(ADP-ribose) synthesis in the extracts using activated DNA (treated with DNAse) and [^32^P]NAD^+^. To this end proteins of the extracts incubated with activated DNA and [^32^P]NAD^+^ were resolved by SDS-PAG electrophoresis (Fig. 7). Lanes 1-5 and 6-10 represent the PAR distribution after separation of the reaction mixtures corresponding to NMR and mouse extracts, respectively. Lanes 11 and 12 correspond to reaction mixtures containing 70 nM recombinant human PARP1 incubated with 40 and 400 µM [^32^P]NAD^+^, respectively. For clarity lanes 13 and 14 represent a low intensity image of lanes 11 and 12. PARP1 synthesizes up to 90% of the total cell PAR in response to DNA damage [40]. The length of PAR, along with other factors, depends upon NAD^+^ concentration [41]. Under saturating concentration of NAD^+^ the PAR length can reach 200-400 ADP units, but it becomes shorter at low micromolar concentration [41]. As expected, the products of PARP1 autoPARylation at 40 µM NAD^+^ appear as a wide smear band (lanes 11 and 13) with an apparent molecular mass over 120 kDa. The products synthesized by PARP1 at 400 µM NAD^+^ spread along the gel (lane 12) and are predominantly concentrated at the border of separating and concentrating parts of the gel (lane 14). It was revealed that, as a whole, the level of PAR synthesis was higher in the extracts of NMR than mouse cells (lanes 1-5 vs lanes 6-10). For NMR cell extract, the PAR synthesis efficiency at 24 h post-irradiation was definitely higher (about 1.5 times) than for other time points, for mouse cell extracts the difference did not exceed 20%.

Along with smeared radioactive bands, several sharp bands of low intensity were observed, particularly the well-defined 95-kDa bands, which were more abundant in mouse cell extracts (lanes 6-10 vs lanes 1-5). For our PARylation test we used the [^32^P] NAD^+^ preparation obtained by ourselves without purification after synthesis. Since our [^32^P] NAD^+^ preparation contained some amount of α[^32^P]ATP that was not converted to [^32^P] NAD^+^ during its synthesis, we proposed that aforementioned sharp bands appeared as a result of α[^32^P]ATP interaction with extract proteins. For instance, ATP-dependent DNA ligases during catalysis transfer AMP moieties on themselves forming rather stable intermediates [42]. To test this proposal, we incubated cell extracts with α[^32^P] ATP and resolved the products side-by-side on the gel with the extracts incubated with [^32^P] NAD^+^. PARP1 and T4 DNA ligase were included as control proteins (Fig. S5). In the ATP containing reaction mixtures several weak radioactive bands were revealed corresponding in electrophoretic mobility to the aforementioned bands appeared in the reaction mixtures with [^32^P] NAD. T4 DNA ligase, which is ATP-dependent, was efficiently labeled when incubated with [^32^P] NAD^+^ preparation (lane 12). Altogether these data may speak in favor of a hypothesis that these bands are related with DNA ligases or their proteolytic fragments rather than with oligo(ADP-ribosyl)ated proteins.

### Affinity labeling of NMR and mouse cell extract proteins with chemically reactive DNA intermediates of the NER and BER systems

**Figure 8:**
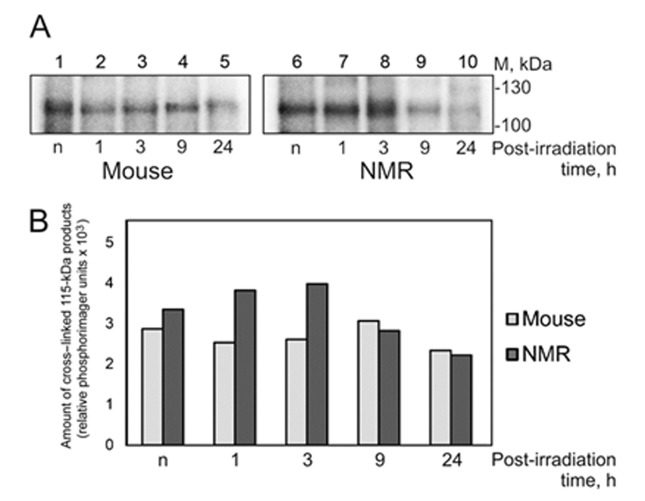
C**ross-linking of NMR and mouse cell extract proteins to dC^FAP^-DNA.** (**A**) The 25 nM [^32^P]dC^FAP^-DNA (54-mer) was incubated with 1.2 mg/ml of mouse (lanes 1-5) or NMR (lanes 6–10) cell extract proteins and buffer components. The mixtures were exposed to UV-light irradiation at 312 nm, 1.5 J/cm^2^·min for 10 min. After the UV-light induced cross-linking the reaction mixtures were treated with benzonase (0.1 unit per 1 μl of the reaction mixture for 30 min at 37ºC). Then the products were analyzed as described in ‘Photocross-linking of cell extract proteins with dC^FAP^-DNA’. ‘n’ denotes the extract of non-irradiated cells; 1–24 designate the extracts of cells cultivated for definite time (in hours) after UVC-irradiation. (**B**) Quantification showing the yield of the 115-kDa cross-linking products.

Affinity labeling as has been shown earlier is a powerful tool in study of protein–nucleic acid interactions in complex systems such as cell extracts [43,44]. Introduction of modifying groups into the structure of DNA probe, for instance mimicking DNA lesions, allows targeting DNA to interaction with proteins of particular way of DNA repair. To study the NER specific proteins in the extracts of NMR and mouse cells we applied 54 bp DNA containing exo-N-[2-[N-(4-azido-2,5-difluoro-3-chloropyridin-6-yl)-3-aminopropionyl]aminoethyl]-2'-deoxycytidine (dC^FAP^). This photoreactive group has been earlier shown to provide high yield of cross-links with proteins [44,45]. DNAs containing dC^FAP^ have high affinity for NER proteins, but are refractory to excision of this lesion by the NER specific endonucleases [28,47,48]. Positioning the radioactively labeled phosphate group nearby the photoreactive lesion, which provides cross-linking to a protein, allows removing the main part of cross-linked DNA by nuclease treatment keeping the label at the protein. Such treatment is necessary to avoid distortion of the apparent molecular mass of modified protein during electrophoretic analysis. One of most intensive products of protein cross-linking in the extracts of NMR and mouse cells with DNA bearing the photoreactive lesion has an apparent molecular mass of 115 kDa (Fig. 8).

This product was revealed in both extracts and UVC-light treatment of the cells did not influence drastically the efficiency of protein cross-linking (Fig. 8, panel B). The intensity of the 115-kDa band was however slightly higher for the extracts of NMR cells. Taking into account the apparent molecular mass of the product we proposed that it is related to PARP1. To confirm this proposal we performed crosslinking without and after preliminary incubation of the samples with NAD^+^ (Fig. S6). Purified human recombinant PARP1 was used as control protein. Disappearance of bands corresponding to cross-linked PARP1 when NAD^+^ was present (compare lanes 1, 2 and 5 with lanes 2, 4 and 6) confirms this proposal. AutoPARylation of PARP1 leads to dissociation of PARP1-DNA complex due to electrostatic repulsion between DNA and PAR [49].

**Figure 9:**
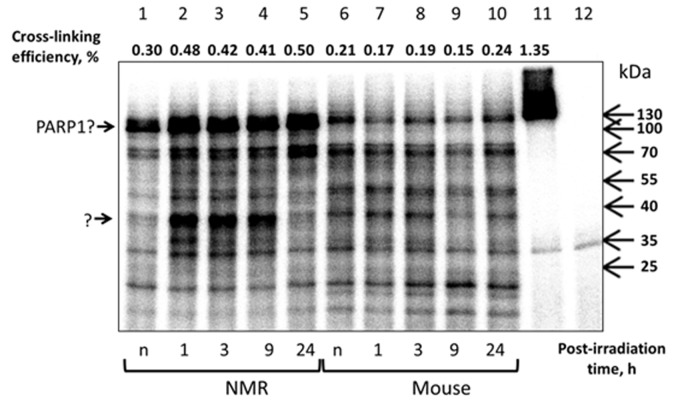
**Cross-linking of NMR and mouse cell extract proteins with AP DNA.** 100 nM 5'-[^32^P] 32-mer AP DNA was incubated with 0.5 mg/ml of NMR (lanes 1–5) or mouse (lanes 6–10) cell extract proteins. The control probes contained buffer components and AP DNA (lane 12) or buffer components, AP DNA and 70 nM human recombinant PARP1 (lane 11). Samples represented in lanes 1 and 6 correspond to the extracts of non-irradiated cells. The reaction mixtures were incubated at 37ºC for 10 min. Then the probes were treated with 20 mM NaBH_4_. The products were then separated and analyzed as described in the section ‘Cross-linking of cell extract proteins with AP DNA’. The intensity of the 120-kDa product estimated as a part of cross-linked DNA (%) is shown at the top of the image.

AP site is a central intermediate arising in the BER process appears as a result of action of various DNA glycosylases or spontaneously [32,33]. AP sites are most abundant among DNA lesions; their level is estimated as 50,000 lesions per cell per day [34]. Deoxyribose in AP sites exists in equilibrium between cyclic furanose and acyclic aldehyde forms and propensity of deoxyribose in AP site to react with amine moieties in its vicinity is well-known. The Schiff base intermediate can be reduced by sodium borohydride (NaBH_4_) or related compounds, forming an irreversible complex between the protein and DNA [32,35]. This makes AP DNA a promising tool in search of cell extract proteins specifically interacting with AP sites in DNAs of different structures [44,50,51]. Patterns of the protein cross-linking to AP DNA are shown in Fig. 9.

Spectra of cross-linked proteins in both extracts are very similar although intensities of some bands are different. The bands with an apparent molecular mass of about 120 kDa having higher intensity in NMR cell extracts (Fig. 9, lanes 1–5 vs lanes 6–10) may be related to PARP1 considering electrophoretic mobility of cross-linked human PARP1 (lane 11). If so, the increased efficiency of PARP1 cross-linking to AP DNA observed for NMR cell extracts (Fig. 9, lanes 1–5) correlates well with the more efficient cross-linking of photoreactive DNA with PARP1 in this extract (Fig. 8B) and higher PARylation activity in NMR cell extracts (Fig. 7, lanes 1–5 vs lanes 6–10). It should be noted that in NMR cell extracts an unknown protein forming the products of an apparent molecular mass of 37 kDa demonstrates rather high increase in cross-linking at the 1 h, 3 h and 9 h points (Fig. 8, lanes 2–4 vs lanes 1, 5) while no such intensive changes were observed for mouse cell extracts (Fig. 8, lanes 6–10).

Taking into account an apparent molecular mass of the cross-linking products and shown by us ability of HMGB1 to interact with cleaved AP site [52], we proposed that aforementioned product may be attributed to HMGB1. HMGB1, non-histone chromatin protein, is known as multifunctional protein having both intracellular and extracellular functions [53]. Due to its ability to interact with DNA HMGB1 is involved in many DNA dependent processes including different pathways of DNA repair [54]. In particular, HMGB1 has been shown to be a co-factor of the BER process [52]. Purified HMGB1 was found to have weak 5ʹ-dRP lyase activity and to stimulate AP endonuclease 1 and FEN1 activities on BER substrates. Further characterization of the roles of HMGB1 in BER demonstrates that the protein acts in regulation of BER sub-pathways by inhibiting single-nucleotide BER and stimulating long-patch BER [55].

To test our proposal we compared cross-linking of cell extract proteins and purified HMGB1 with AP DNA, containing two AP sites situated opposite each other in complementary DNA chains (see Table S3 for DNA structures). This AP DNA was used since it provides considerably more efficient cross-linking to HMGB1 as compared to AP DNA with single AP site (Fig. S7A, lanes 2 and 1, respectively). Products of AP DNAs cross-linking to HMGB1 have analogous bands in the patterns of the extracts (compare lanes 1 and 2 with lanes 3-6). For both organisms the ratios of intensities of cross-liking products for AP DNA with bistranded AP sites in the extracts prepared from non-irradiated cells (Fig. S7B, lanes 2 and 4) and cells cultivated for 3 h after UVC-irradiation (Fig. S7B, lanes 3 and 5) resemble those for AP DNA with single AP site (Fig. 9, lanes 1, 3 and lanes 6, 8). Altogether, the data on AP-DNA cross-linking is in agreement with proposal that the 37 kDa product may be related with HMGB1. Interestingly, that for the NMR cell extracts with higher amounts of the cross-linking products attributed to HMGB1 (lanes 2-4 in Fig. 9) the higher level of strand-displacement DNA synthesis, which is characteristic for long-patch pathway of BER, is observed (lanes 8, 10, 12 vs lanes 6, 14 in Fig. 6). Thus, these findings are in agreement with reported in [55] stimulatory influence of HMGB1 onto long-patch pathway of BER.

## DISCUSSION

In mammalian cells, regulation of DNA repair mechanisms is controlled at multiple levels. Most of the genes encoding repair proteins are not transcriptionally regulated in response to damage, but are expressed constitutively and regulated by posttranscriptional modifications [for review see 19,56], but several NER proteins encoding genes including *Ddb2, Xpc, Xpf,* and *Xpg* known to be induced under genotoxic stress. At the same time the transcription level change depends both on the type of cells and on the type of genotoxic effect. Mammalian cells express genes encoding proteins involved in DNA repair at the well detectable basal level, which provides repair of the damages resulted from endogenous genotoxic actions [19]. Even a relatively slight up-regulation of transcription above the basal level after UV-light treatment (≈ 2-fold) may significantly improve the repair capacity. The observed in the present work time-dependence of post-UV-light exposure mRNA content suggested a homeostatic type of transcription regulation after genotoxic impact. This type of regulation is characterized by a moderate decrease in mRNA content relative to the basal level and its subsequent increase. It might be generally important for repair proteins whose expression above a critical level in principle can be deleterious [19]. In mouse and human fibroblasts the increase in expression level of *Xpf* and *Xpg* after UV-light irradiation has been reported earlier [27,57]. A substantial increase in *Xpg* mRNA level was detected in mouse (up to 6 times). The mechanism of NER system catalyzing a specific excision includes a coordinated action of multiprotein ensembles of variable composition. In these complexes, protein factors and enzymes specifically interact with each other and with DNA, which provides an additional control over the specificity and accuracy of removal of the damaged part of DNA [58,59].

Induction of APE1 mRNA accompanied by the increase of AP-site cleavage activity in response to treatment of CHO (Chinese hamster ovary) cells with H_2_O_2_ or NaOCl has been demonstrated earlier [60]. APE1 induction (both mRNA and protein) and increase in activity were observed at 3-9 h after treatment (with 3 h being the earliest time point), followed by slow drop to the basal level at 25 h after treatment. Interestingly, a more pronounced increase in activity in our experimental system corresponded to 1-9 h, whereas the 3 h point demonstrated minimal mRNA level. It should be noted that in our system UVC-light irradiation was used as a genotoxic stimulus. An analogous APE1 induction by reactive oxygen species has been demonstrated for human cells (HeLa and primary fibroblasts) [61].

Several examples of up-regulation of the mRNAs encoding the BER-related proteins have been summarized in the review [19]. Up-regulation of mRNAs synthesis in response to DNA damage was reported for XRCC1, Polβ, and some DNA glycosylases recognizing oxidized nucleobases. Interestingly, that in some cases transcriptional up-regulation of the BER-related proteins was caused by UVC-light irradiation along with the agents, which causes DNA damages repaired by BER. This concerns flap endonuclease 1 (FEN1) [62,63], DNA ligase I [64], and N-methylpurine DNA glycosylase [65]. It should be noted that FEN1 and DNA ligase I, which are involved in LP BER, are mainly associated with DNA replication. Interestingly, there is no convincing evidence that up-regulation of mRNA expression of either of the aforementioned BER genes brings about the increase of overall BER capacity. In most cases (with exception of DNA synthesis) the efficiency of DNA repair-related reactions are higher in NMR cell extracts than in mouse ones that is in agreement with the proposal of enhanced DNA repair capacity in long-lived organisms based on higher expression of some genes essential for DNA repair [17].

Stimulation of DNA synthesis in the long-patch pathway may be mediated by HMGB1 [52,55] and more effective DNA synthesis characteristic for NMR cell extracts obtained from cultures at 1, 3 and 9 h after irradiation is in line with our proposal of the higher content of HMGB1 in these extracts as determined by cross-linking experiments.

A higher activity of PARylation system in NMR cells demonstrated here is in agreement with earlier published data, where an enhanced synthesis of PAR in the cells of long-lived organisms has been reported ([66–68] and references therein). It should be stressed that NMR cells were not included in analysis of PARylation system in the aforementioned studies. Moreover, to our knowledge, no data concerning time-dependent changes of PARylation activity in cells after exposure to DNA damaging agents have been reported yet. It would be of interest to establish how the activity of the cellular PARylation system varies with time after exposure to DNA damage agents causing DNA lesions repaired by the BER system.

Paradoxically, the extracts of NMR cells demonstrated higher activity in the BER and NER related reactions, but NMR fibroblasts were more sensitive to H_2_O_2_ and UV light as compared to mouse fibroblasts [20]. This discrepancy may be explained by more effective PARylation system in NMR cells. Upon action of genotoxicants on the cells higher efficiency of PAR synthesis may result in depletion of cellular NAD^+^/ATP pool bringing about cell death. Indeed, several studies based on different cell models demonstrated that targeted siRNA or pharmacological inhibition of PARP protected cells from H_2_O_2_-induced death [69–73]. At the same time, under normal conditions higher activity of PARylation system can contribute to more effective maintenance of genome integrity.

Cumulatively, more efficient excision repair and higher activity of the PARylation system appear to contribute to the delay of senescence and formation of neoplasias in NMR.

## MATERIALS AND METHODS

### Materials

Synthetic oligonucleotides were obtained from Laboratory of Biomedicinal Chemistry (ICBFM SB RAS, Russia). [γ-^32^P] ATP (3000 Ci/mmol), [α-^32^P] ATP (3000 Ci/mmol) and [α-^32^P] dCTP (3000 Ci/mmol) were from Laboratory of Biotechnology (ICBFM SB RAS, Russia). T4 polynucleotide kinase, *E. coli* uracil DNA glycosylase, T4 DNA ligase were from Biosan, Russia. The plasmids containing cDNA of human apurinic/apyrimidinic endonuclease 1 (APE1) and rat DNA polymerase β (Pol β) were a kind gift of Dr. S.H. Wilson (National Institute of Environmental Health Sciences, NIH, NC, USA). Recombinant APE1 and Pol β were purified as described previously [74,75]. FAP-dCTP was synthesized as described [44]. Proteinase K and benzonase were from Merck and Novagen, respectively. Protein markers were from Bio-Rad. Cell proliferation & toxicity assay (EZ4U Assay) was from Biomedica.

### Cell cultivation

The establishment of *H. glaber* cell culture NSF8 derived from skin was described [10]. Here *H. glaber* cells were cultured in alpha MEM supplemented with 15% of FBS (Gibco), 10% AmnioMAX II Complete Medium (Gibco), 5 ng/ml bFGF, 10^5^ U/L penicillin, and 100 mg/L streptomycin, 2.5 mg/L amphotericin B at 32°C in conditions of 5% CO_2_.

Primary fibroblast *M. musculus* cell line were established in the Laboratory of Animal Cytogenetics, the Institute of Molecular and Cellular Biology, Russia, from a female embryo that was partially digested with 0.25% trypsin with 0.2% EDTA [76]. Digested tissues were cultured in alpha MEM supplemented with 15% of FBS (Gibco), 10^5^ U/L penicillin, and 100 mg/L streptomycin, 2.5 mg/L amphotericin B at 37°C in conditions of 5% CO_2_.

All cell lines were deposited in the IMCB SB RAS cell bank (“The general collection of cell cultures”, Nº 0310-2016-0002).

### UVC-light irradiation

Cells were grown to 70–80% confluence, media was aspirated and the cells were rinsed with PBS, after which a thin layer of PBS was added and cells were exposed to UVC light (254 nm) (Lamp Philips, Germany) at a dose rate of 1.25 J/m^2^●sec as described in [77]. After UVC irradiation PBS was aspirated and the fresh medium was added to further cells cultivation (for 1, 3, 9 or 24 h). Then cell were harvested and used to prepare whole-cell extracts or isolate RNA. The metabolic activity/viability of cells at the time points after irradiation that then were used for the tests of enzymatic activities and evaluation of mRNA levels was tested using cell proliferation assay EZ4U (Biomedica) as recommended by manufacturer.

### Whole-cell extracts preparation

*H. glaber* and *M. musculus* cells were used to prepare whole-cell extracts as described in [78]. Extracts were aliquoted and stored at -70°C. The cells aliquots for RNA isolation were placed in RNA–Later and stored at -70ºC until RNA isolation.

### Quantitative Real-time PCR (for mRNA encoding NER related proteins)

Total RNA was isolated using an Aurum Total RNA Mini Kit (Bio-Rad). The quality and quantity of the isolated RNA were assessed in an Agilent 2100 Bioanalyzer (RIN ≥ 7 for all samples) according to the manufacturer’s instructions. RNАse-free DNase I (Fermentas) was used for DNase treatment according to the manufacturer’s protocol. A High Capacity cDNA Archive Kit (Applied Biosystems) was used to synthesize the cDNA. The levels of target genes expression were assessed by quantitative PCR in real time using the M-436 kit for real-time PCR in the presence of SYBR Green I (Sintol) in an ABI PRISM 7000. The primers for the target and housekeeping genes (Table S1, Supporting information) were designed using the Primer Express^®^ software v. 2.0 (Applied Biosystems) and Primer-BLAST software. The reaction was conducted according to the manufacturer’s protocol and the reaction conditions were the same for all primer pairs.

### RT-qPCR (for mRNA encoding BER related proteins)

Reverse transcriptase quantitative PCR was performed under universal conditions (RT step: 45ºC 1800 s, 95ºC 300 s; amplification step 95ºC 10 s, 61ºC 10 s, 72ºC 10 s, 84ºC 5 s) with Roche Lightcycler 96. The reaction was performed in a final volume of 15 µl containing 5 ng (3.75 µl) of total RNA, 3.75 µl of primer mixture (final concentration of 500 nM each) and 7.5 µl of universal RT-PCR master mix with SYBR Green I (BiolabMix) using one tube protocol. The primers used in this study are listed in Table S2, Supporting Information. The testing algorithm included a number of controls: positive reference controls to detect any variation between runs, and no-template controls.

### The 5ʹ-end labeling and purification of oligodeoxyribonucleotides

Oligodeoxyribonucleotides were the 5'-^32^P-phosphorylated with T4 polynucleotide kinase and purified by 20% polyacrylamide 7.0 M urea gel electrophoresis as described [79] followed by electro-elution and precipitation with 2% solution of LiClO_4_ in acetone. The precipitated oligodeoxyribonucleotides were dissolved in 10 mM Tris-HCl (pH 8.0) and 1 mM EDTA.

### Synthesis of the extended linear DNA duplexes

Extended DNA duplexes bearing modifying groups were synthesized essentially as described [46,80]. To synthesize an effective NER substrate, nFlu-modified oligonucleotide with length corresponding to 16 mer unmodified oligonucleotide was enzymatically ligated with flanking oligonucleotides (see Table S3, Supporting Information) to yield single-stranded 137 nt DNA. To synthesize 54 nt DNA probes bearing Fap-dC-photoactivatable damage and [^32^P] at the internal positions of DNA chain the chemo-enzymatic approach using DNA polymerase β and T4 DNA ligase and T4 polynucleotide kinase was applied. The modified DNAs were purified using PAG electrophoresis under denaturing conditions [79]. After spectrophotometric quantification, the modified ssDNA were annealed with the appropriate complementary unmodified strand.

### [^32^P] NAD^+^ synthesis

The synthesis of radioactive NAD^+^ was carried out from α−[^32^P]-ATP according to [81] with modification described in [82]. The reaction mixtures containing 1 mM ATP, 10 MBq of α−[^32^P]-ATP, 20 mM MgCl_2_, 2 mM β-nicotinamide mononucleotide, and 5 mg/ml nicotinamide nucleotide adenylyltransferase in 25 mM Tris-HCl, pH7.5 were incubated at 37ºC for 60 min, then stopped by heating up to 90ºC for 3 min. After removal of denatured protein by centrifugation the solution was used as a reactant.

### *In vitro* NER assay

The efficiency of removal of DNA region containing model lesions was determined using the 3ʹ-end-labeling (or “fill-in” synthesis) method [83] with several modifications. The reaction mixtures (usually 30 μl) containing 600 fmol of model DNA duplex, 500 nM template 5ʹ-gggggctcggcaccgtcaccctggatgctgtagg-p-3ʹ and NER-competent cell extract (the amounts of proteins and extract type specified in the figure legends) in 1x NER buffer (25 mM Tris-HCl, pH 7.8, 45 mM NaCl, 4.4 mM MgCl_2_, 0.1 mM EDTA, 4 mM ATP) were incubated for 15-60 min at 30°C. After incubation the mixture was heated to 95ºC and slowly cooled down to room temperature for annealing of excised oligonucleotide to specific template. 10xTaq-polymerase buffer (1/10 of reaction mixture volume), Taq polymerase (5-10 units) and α-[^32^P]-dCTP (500-600 Bq) were added and the probes were incubated for 5 min at 37ºC, then 1.2 µl of dNTP mixture containing 100 µM dATP, dGTP, dTTP and 50 µM dCTP was added and incubation was continued for 15 min. The reaction was stopped by adding proteinase K solution (0.5 µl, 4 mg/ml). After 60 min of proteinase treatment at 37ºC the reaction mixtures were ethanol precipitated and analyzed by electrophoresis in polyacrylamide gel under denaturing conditions [79]. The gels were dried and subjected to autoradiography for quantification using Typhoon FLA 9500 (GE Healthcare) and software (Quantity One). Quantitative analysis was performed relatively to total radioactivity in each lane, using control lane signal as baseline.

### Uracil excision activity of cell extracts

Reaction mixtures (10 µl) containing 100 nM 5'-[^32^P] 32-mer U-DNA, 50 mM Tris-HCl (pH 8.0), 40 mM NaCl, 10 mM EDTA, 1 mM DTT, 0.1 mg/ml BSA were assembled on ice. Cell extracts at final protein concentration of 0.5 mg/ml or 5 u/µl of UDG were added as indicated in the figure legends. The reaction mixtures were incubated at 37ºC for 10 min. Then reaction mixtures were supplemented with 10 mM NaOH and loading buffer and heated at 97ºC for 10 min followed by product analysis by denaturing electrophoresis in 20% polyacrylamide gel [79]. Further analysis and quantification was done as described in the previous section.

### AP-site cleavage activity of cell extracts

AP DNA was prepared by incubation of 1 µM U-containing DNA with 10 u/μl UDG for 30 min at 37ºC immediately before experiment. Reaction mixtures (10 µl) containing 100 nM 5'-[^32^P] 32-mer AP DNA, 50 mM Tris-HCl (pH 8.0), 40 mM NaCl, 8 mM MgCl_2_, 1 mM DTT, 0.1 mg/ml BSA were assembled on ice. Cell extract proteins at a final protein concentration of 0.5 mg/ml or 10 nM APE1 were added as indicated in the figure legends. The reaction mixtures were incubated at 37ºC for 10 min. Then AP sites were stabilized by treatment with 10 mM NaBH_4_ at 0ºC for 30 min. The mixtures were supplemented with loading buffer, heated at 97^0^C for 10 min and the products were analyzed as described in the previous section.

### DNA polymerase activity of cell extracts

Reaction mixtures (10 µl) containing 100 nM 5'-[^32^P] 32-mer DNA (5'-dRP-DNA or 5'-pDEG-DNA (see Table S4), 50 mM Tris-HCl (pH 8.0), 40 mM NaCl, 8 mM MgCl_2_, 1 mM DTT, 0.1 mg/ml BSA were assembled on ice. Cell extracts at final protein concentration of 0.5 mg/ml or 10 nM Pol β were added as indicated in the figure legends. The reaction mixtures were incubated at 37ºC for 10 min and the products were analyzed as described in the previous section.

### PARylation of cell extract proteins

Reaction mixtures (10 µl) containing 0.6 A260/ml of activated DNA, 40 or 400 µM [^32^P]-NAD^+^, 100 µM α-[ [^32^P]-ATP (when indicated), 50 mM Tris-HCl (pH 8.0), 40 mM NaCl, 8 mM MgCl_2_, 1 mM DTT, 0.1 mg/ml BSA were assembled on ice. Cell extracts at final concentration of protein 0.5 mg/ml or 70 nM PARP1 or 50 nM T4 DNA ligase were added as indicated in the figure legends. The reaction mixtures were incubated at 37ºC for 4 min. The mixtures were supplemented with loading buffer, heated at 97ºC for 10 min and the products were further analyzed by SDS-PAG electrophoresi [80].

### Cross-linking of cell extract proteins with AP DNA

Reaction mixtures (10 µl) containing 100 nM 5'-[^32^P] 32-mer AP DNA, 50 mM Tris-HCl (pH 8.0), 40 mM NaCl, 20 mM EDTA, 1 mM DTT, 0.1 mg/ml BSA were assembled on ice. Cell extracts at final protein concentration of 0.5 mg/ml or 70 nM PARP1 were added as indicated in the figure legends. The reaction mixtures were incubated at 37^0^C for 10 min. Then reaction mixtures were treated with 10 mM NaBH_4_ 0^0^C for 30 min. The mixtures were supplemented with loading buffer and heated at 97^0^C for 10 min and the products were analyzed by SDS-PAG electrophoresis [80]. The gels were dried and subjected to autoradiography for quantification using Typhoon FLA 9500 (GE Healthcare) and software (Quantity One).

### Photocross-linking of cell extract proteins with dC^Fap^-DNA

Photocross-linking was performed in the reaction mixture (10–30 μl) containing photoreactive DNA (25 nM) and the proteins (1.2 mg/ml) in the buffer for modification (25 mM Tris-HCl, pH 8.0, 5 mM MgCl_2_, 25 mM KCl, 0.125 mM β-mecaptoethanol). Samples were exposed to UV irradiation (312 nm) in a BIO-LINK®BLX (Vilber Lourmat) for 15 min at 1 J/cm^2^●min. Autopoly(ADP-ribosyl)ation of PARP1 (when indicated in the figure legend) was performed in the presence of 1 mM NAD^+^ for 30 min at 37°C. After that the reaction mixtures were treated with benzonase (0.1 unit/μl of reaction mixture, for 30 min at 37°C). The mixtures were supplemented with loading buffer, heated at 97^0^ for 10 min and the products were further analyzed as described in the previous section.

## Supplementary Material

Supplementary File
