# Supplementary material for: Naked mole rat cells display more efficient excision repair than mouse cells
Source: Aging (Albany NY). 2018 Jun 20;10(6):1454–73. doi: 10.18632/aging.101482 (PMC6046242; doi:10.18632/aging.101482)
Supplement: Supplementary File [file aging-10-101482-s001.pdf]

SUPPLEMENTARY MATERIAL

Table S1. Primers (for the NER system).

Organism	Gene	For, Rev*	Sequence (5′→3′)
Naked mole rat	*Tubb2a*	For	CAGACTCACGGACACAGACC
		Rev	GTACACGTTGATTCTCTCCAGC
	Tubb1	For	AAAAGTATGTGCCCCGAGC
		Rev	CACTCTCACTCCTCACCACG
	Polr1b	For	TCTTGCTACTGGGAATCTGCG
		Rev	TTACGGGACAAAGGAAGCCC
	Ddb2	For	TTCCCTACGAGTCAAGGACAG
		Rev	TCTTCACTGGCTCTCGGTC
	Xpc	For	GGTTGATGCTGAGTGGTGGG
		Rev	TCTCTGGGTAGATGGCCTCG
	Ercc2 (xpd)	For	TTCCTGGGTTTCCTGAGACG
		Rev	GTGGAGAGAAGTCAGCGAGG
	Ercc4 (xpf)	For	TAGCAAGAGGCACAGCATCG
		Rev	CGCACATTTCTGGTGTCAGG
	Ercc5 (xpg)	For	CAGACCTCGGGAACCATC
		Rev	TCCTTCCGTGTAATCACTGC
House mouse	*Tubb2a*	For	GCCTCCACCCCTTCTACAAC
		Rev	TGTTGCCAGCAGCTTCATT
	Polr1b	For	CGCTACCTCTCCCATTTCCG
		Rev	AGGCTGGAATAGATGCCGTG
	Actb	For	ACATGGCATTGTTACCAACTGG
		Rev	CCTGGATGGCTACGTACATGG
	Ddb2	For	TTAAAGGGATTGGAGCTGGAG
		Rev	GCTCTTGGCAGAAACATCAAG
	Xpc1	For	CCTTGAGACCCTATCGGAGC
		Rev	CTTCTCCACGACAATACCCG
	Ercc2	For	TTCTGCTCTCAGTGGCTCG
		Rev	GCGCATAGCATCAAAGGTG

	Ercc4 (Xpf)	For	AGCATGGTTGTCCCCGA
		Rev	CTATGTCAATACCCCGCCG
	Ercc5 (Xpg)	For	GGCGCGTCCTTTATCCTAAC
		Rev	CGGACTCCTTTAAGTGCTTGG

*For and Rev designate forward and reverse primers, respectively.

Table S2. Primers (for the BER system).

Organism	Gene	For, Rev*	Sequence (5′→3′)
Naked mole rat	*Actb*	For	TCGCTCTCCACCTTCCAGCA
		Rev	AGGGTGAAAGGCAGCGAAGTA
	Tubb	For	TCCTCGCGCTATTTTGGTGGA
		Rev	GTTGTTGCCTGCCCCAGACT
	Gapdh	For	TGTTCACCACCATGGAAAAGGC
		Rev	CAAACATGGGGGCATCCGC
	Apex1	For	CTGGTCAGCGCCTTCTGACA
		Rev	CCGGCCTTCCTGGTCATGTT
	Parp1	For	AGGCAGCGGACAAGCTCTAC
		Rev	ATGGGTGACTGCACCATGAG
	Parp2	For	AGAAGATGATGCCCGGAGAA
		Rev	GATTTCCTTGGCCTTGGTGA
	Polb	For	ATGAGCAAACGGAAGGCGCCGCAGG
		Rev	GTTCCTACTCCAGGCAATTTCTTAGCTT
	Xrcc1	For	AGCTGGGTGCCAAGTATCGG
		Rev	ACCCACTCTTTGCGCACGAT
	Lig3	For	CCCCTGTGAAACTGGGAGAG
		Rev	CAGCAGCACCTTCACCTTTG
House mouse	*Actb*	For	CACTGTCGAGTCGCGTCC
		Rev	ACGACCAGCGCAGCGATA
	Tubb	For	TTGGGAGTCCTCTTTCAGCC
		Rev	ACATTTTCGATCAGCTCCGC
	Gapdh	For	CCTGCACCACCAACTGCTTA
		Rev	AGTGATGGCATGGACTGTGG
	Apex1	For	GGAGCCTAAGGGCTTTCGTC
		Rev	CTTTGCTGCCCCCTTACTCT
	Parp1	For	ATGGTGCAGTCACCCATGTTC
		Rev	CAGAGAAGCCATCCACCTCA
	Parp2	For	CAGGCTGAGTAACTGGGTGG
		Rev	TTTAGGCGAGAGGCAAAGCA
	Polb	For	TCCCAGCGAGAAGGATGGAAA

		Rev	GGGCGGATGGTGTACTCATTG
	Xrcc1	For	CCCAACCGTGTTCGCATTTT
		Rev	TGGCGAGTCCTTGCTGTATG
	Lig3	For	TCACTGGGGTGCGGCTCTAC
		Rev	GTGGCTGAGCCCATGTCAAAT

*For and Rev designate forward and reverse primers, respectively.

Table S3. DNA substrate and DNA probe for photo-crosslinking.

DNA duplex	DNA duplex structure
DNA substrate<br>137 bp;<br>**X** – nFlu	5′-TGGACGATATCCCGCAAGAGGCCCGGCAGTACCGGCATAACCAAGCCTA<br>3’-ACCTGCTATAGGGCGTTCTCCGGGCCGTCATGGCCGTATTGGTTCGGAT<br>TGCCTACAGCATCCAGGG**X**GACGGTGCCGAGGATGACGATGAGCGCATTGTT<br>ACGGATGTCGTAGGTCCCGCTGCCACGGCTCCTACTGCTACTCGCGTAACAA<br>AGATTTCATACACGGTGCCTGACTGCGTTAGCAATT-3’<br>TCTAAAGTATGTGCCACGGACTGACGCAATCGTTAA-5’
DNA probe<br>54 bp<br>**Y** – Fap-dC<br>**p** – ^{32}P	5’-AAGCCTATGCCTACAGCATCCAGGG-**Yp**GACGGTGCCGAGGATGAAC<br>3’-TTCGGATACGGATGTCGTAGGTCCC-G-CTGCCACGGCTCCTACTTG<br>GATGGCGCA-3’<br>CTACCGCGT-5’

Table S4. DNA substrates for the BER system activity and AP DNA cross-linking.

DNA duplex	DNA duplex structure
U-DNA	5′-GGGAGGCCCTGGCGTT-**U**-CCCGGCTTAGTCGCC-3′<br>3′-CCCTCCGGGACCGCAA-**G**-GGGCCGAATCAGCGG-5′
AP DNA	5′-GGGAGGCCCTGGCGTT-**O**-CCCGGCTTAGTCGCC-3′<br>3′-CCCTCCGGGACCGCAA-**G**-GGGCCGAATCAGCGG-5′
	5′-GGGAGGCCCTGGCGTT-**O**-CCCGGCTTAGTCGCC-3′<br>3′-CCCTCCGGGACCGCAA-**O**-GGGCCGAATCAGCGG-5′
dRP-DNA	5′-GGGAGGCCCTGGCGTT **dRP**-CCCGGCTTAGTCGCC-3′<br>3′-CCCTCCGGGACCGCAA--**G**--GGGCCGAATCAGCGG-5′
pDEG-DNA	5′-GGGAGGCCCTGGCGTT **pDEG**-CCCGGCTTAGTCGCC-3′<br>3′-CCCTCCGGGACCGCAA---**G**--GGGCCGAATCAGCGG-5′

AP DNAs were produced by treatment of 1 µM U-DNA with 1 u/µl *E. coli* UDG for 30 min at 37°C. dRP-DNA was produced by treatment of 1 µM AP DNA with 10 nM human APE1 for 10 min at 37°C. **U**, dRP, pDEG, and O designate uracil, 5′ deoxyribose phosphate, 5′ diethyleneglycol phosphate and abasic site, respectively.

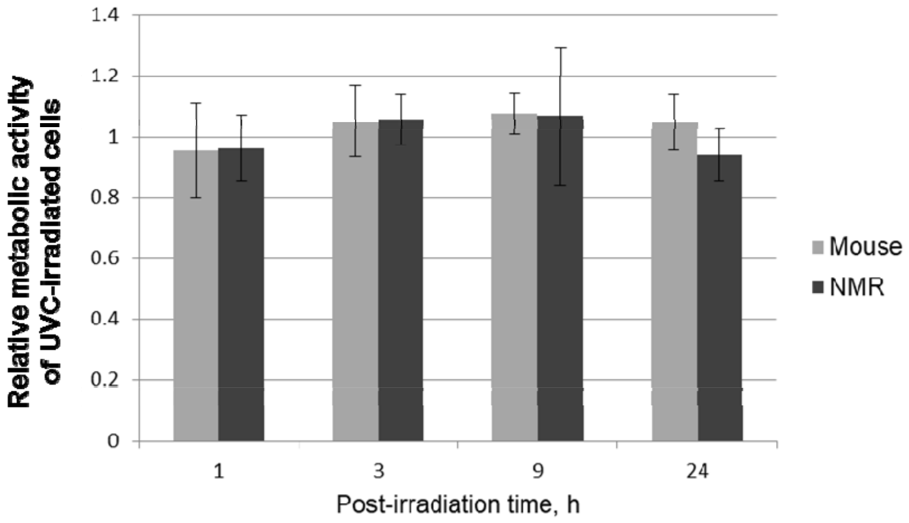

Figure S1. **Relative metabolic activity of UVC-irradiated cells.** The metabolic activity was measured after 1, 3, 9 and 24 h after irradiation using cell proliferation assay EZ4U (Biomedica) as recommended by manufacturer. For each cell line the metabolic activity of cells at definite post-irradiation time was normalized to that of untreated cells. The data are the mean ±SD, n=3.

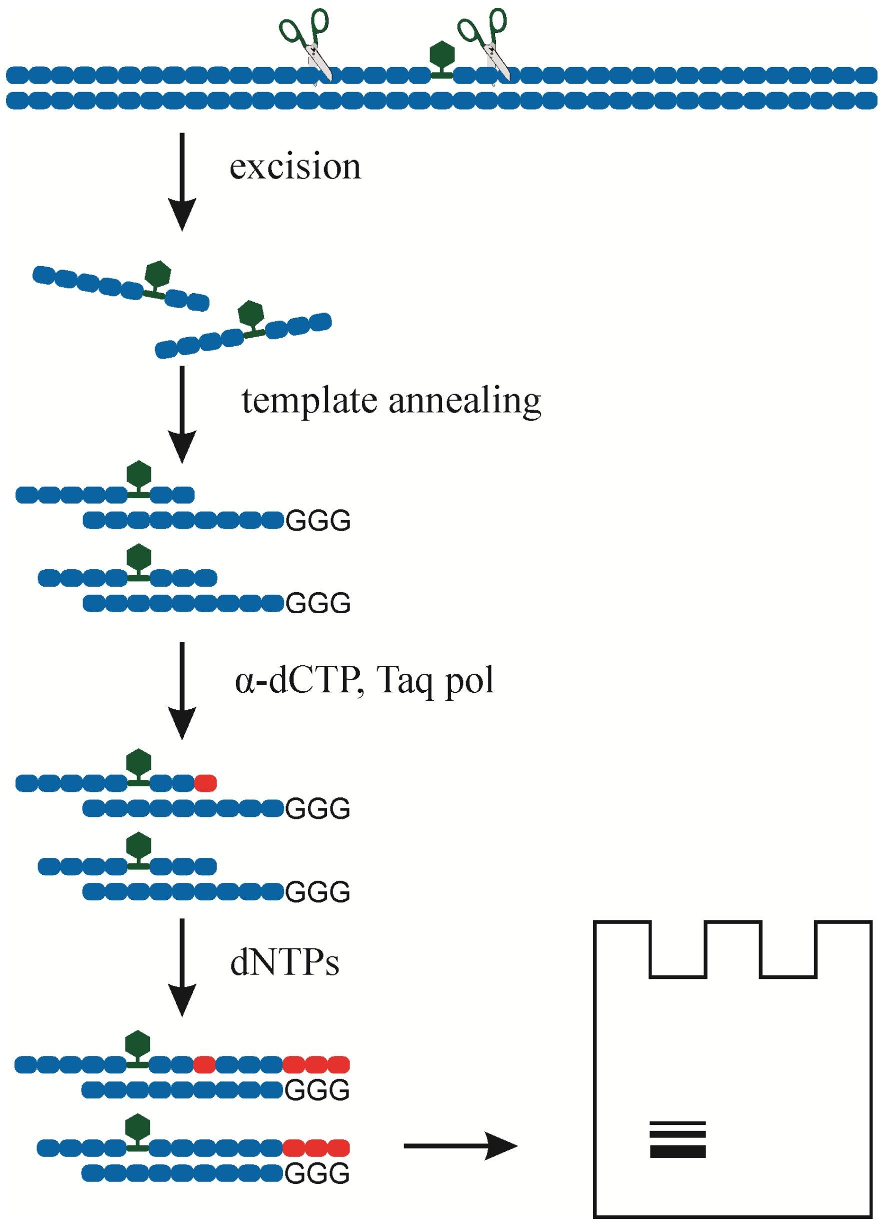

Figure S2. **Flowchart of NER in vitro assay.** Green hexagon represents a bulky modification; red ovals, radioactive label; dark blue ovals, the sequence of specific template.

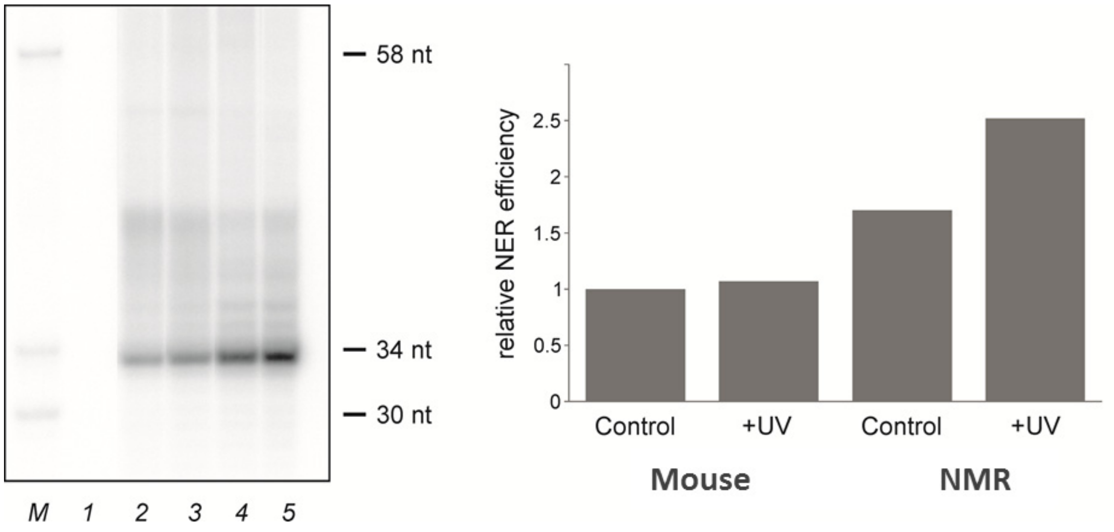

Figure S3. NER excision activity of mouse (lanes 2 and 3) and NMR (lanes 4 and 5) cells extract on model DNA containing artificial damage nFlu. UVC-irradiated cells collected at 24 h after irradiation (lanes 3 and 5) or non-irradiated cells (control cells (lanes 2 and 4) were used for cell extract preparation. The model DNA was incubated for 45 min at 30°C with cell extracts (15 nM model DNA, 0.3 mg/ml extract proteins). The excision products were detected by annealing to specific template containing 5′-GpGpGpGpG overhang, which served for the following end-labeling using α-[^{32}P]-dCTP and Taq DNA polymerase. The reaction products were resolved on a 10% denaturing polyacrylamide gel. ^{32}P-labelled oligonucleotides were used as size markers (lane M). nFlu-DNA without cell extract was used as a negative control (lane 1). The lengths of marker DNA are shown on the right side of autoradiogram. Quantification of the excision products shown in lanes 2-5; the excision efficiency of the extracts was normalized to that of non-irradiated mouse cells extract (lane 2).

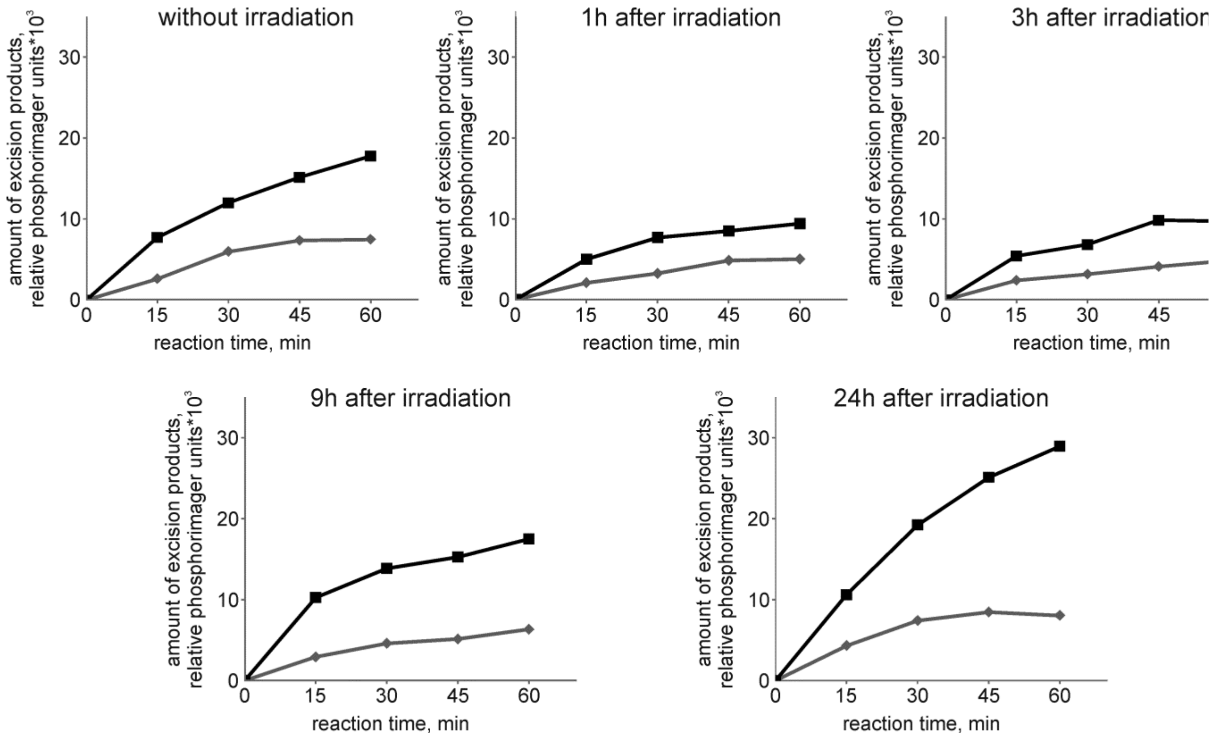

Figure S4. Relative NER excision activity of mouse (gray line) and NMR (black line) cell extracts on model DNA containing artificial damage nFlu. The excision efficiency of each cell extract was measured at 15, 30, 45 and 60 minutes of incubation with model DNA (15 nM model DNA, 0.3 mg/ml extract proteins). The excision efficiency of NMR cells extract at 45 minutes was further taken as a unit of activity.

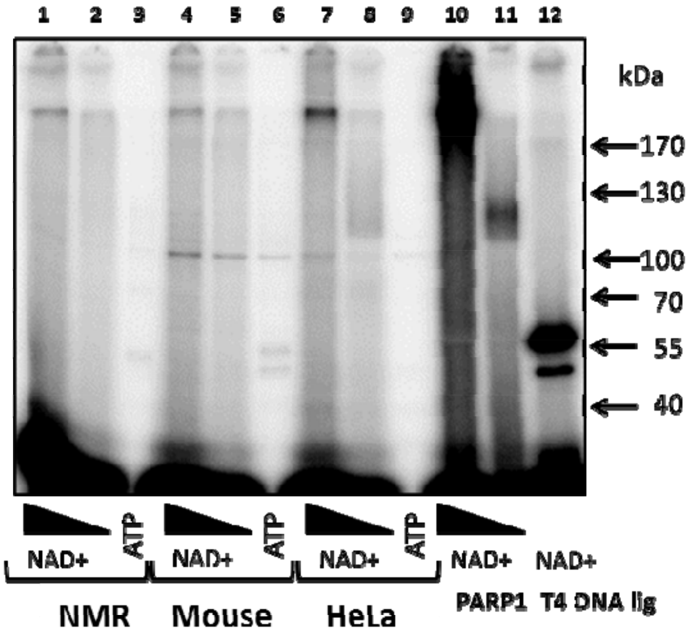

Figure S5. PARylation vs adenylation of proteins. Activated DNA (0.6 A_{260}/ml) in buffer: 50 mM Tris-HCl (pH 8.0), 40 mM NaCl, 8.0 mM $MgCl_2$, 1 mM DTT, 0.1 mg/ml BSA, 400 or 40 μM [^{32}P]NAD+ or 100 μM [^{32}P]ATP was incubated with 0.5 mg/ml of cell extracts of NMR (lanes 1–3) or mouse (lanes 4–6), or HeLa (lanes 7–9) cells. The control probes (lanes 10–12) contained 70 nM PARP1 (lanes 10, 11) or 50 nM T4 DNA ligase (lane 12) instead of the cell extracts. The reaction mixtures were incubated at 37°C for 4 min. The mixtures were then supplemented with loading buffer and heated at 97°C for 10 min and the products were analyzed by SDS-PAGE.

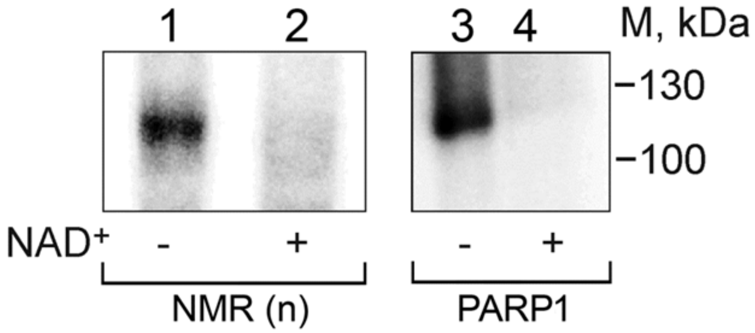

Figure S6. Influence of NAD+ on the cross-linking of NMR extract proteins to dC^{FAP}-DNA. The 25 nM [^{32}P]dC^{FAP}-DNA (54-mer) was incubated with 1.2 mg/ml extract proteins of NMR non-irradiated cells (lanes 1, 2) or 50 nM PARP-1(lanes 3, 4), 1 mM NAD+ (lanes 2, 4) and buffer components. The mixtures were exposed to UV irradiation at 312 nm, 1.5 J/cm2·min for 10 min. After the UV-light induced cross-linking of dC^{FAP}-DNA the reaction mixtures were incubated with benzonase (0.1 u/µl of a sample for 30 min at 37°C). Then the mixtures were treated and analyzed as described in 'Photocross-linking of cell extract proteins with dC^{Fap}-DNA'. 'n' denotes the extract of non-irradiated cells.

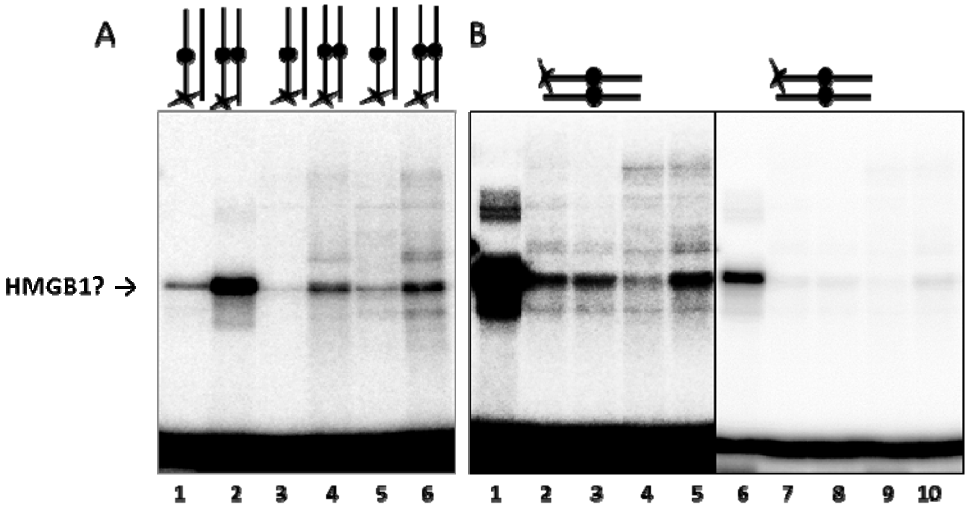

Figure S7. Cross-linking of NMR and mouse cell extract proteins to DNA with single and clustered AP sites. (**A**) Comparison of cross-linking of AP DNA containing single AP site (odd lanes) or AP DNA with two AP sites situated opposite each other (even lanes) to the proteins. 100 nM 5'-[^{32}P] 32-mer AP DNAs was incubated with 2 µM HMGB1 (lanes 1 and 2), or 0.5 mg/ml of mouse (lanes 3 and 4) or NMR (lanes 5 and 6) cell extract proteins and buffer components: 50 mM Tris-HCl (pH 8.0), 40 mM NaCl, 10 mM EDTA, 1 mM DTT, 0.1 mg/ml BSA. Samples represented in lanes 3–6 correspond to the extracts prepared from the cells cultivated for 9 h after irradiation. (**B**) Comparison of cross-linking of AP DNA containing bistranded AP sites with NMR or mouse cell extract proteins and HMGB1. The experiment was performed as described in A, but concentration of HMGB1 was 5 µM and the extracts were prepared from non-irradiated cells (lanes 2 and 4) or the cells cultivated for 3 h after UVC irradiation. Lanes 2 and 3 correspond to mouse and lanes 4 and 5 to NMR cell extracts, respectively. Lanes 6-10 – low intensity image of lanes 1-5. Types of AP DNA are schematically shown at the top; black ovals and asterisks designate AP site and the 5'end radioactive label, respectively.
